# Intraoperative hypotension is not associated with adverse short-term postoperative outcomes after esophagectomy in esophageal cancer patients

**DOI:** 10.1186/s12893-020-01015-z

**Published:** 2021-01-02

**Authors:** Ephraim Teffera Yeheyis, Seyoum Kassa, Hiwot Yeshitela, Abebe Bekele

**Affiliations:** Division of Cardiothoracic and Vascular Surgery, Department of Surgery, School of Medicine, Addis Ababa University, Addis Ababa, Ethiopia

**Keywords:** Esophageal cancer, Esophagectomy, Low blood pressure, Mortality, Anastomotic leak

## Abstract

**Background:**

The effect of low systolic blood pressure and its subsequent postoperative outcome during esophagectomy for esophageal cancer is not well studied.

**Methods:**

A prospective study was conducted and data were collected on patients who underwent esophagectomy and esophagogastric anastomosis for esophageal cancer. Intraoperative hypotension (IOH), defined as systolic blood pressure (SBP) < 90 mm Hg lasting more than 5 min, was recorded. Patients’ 30 days post-operative composite outcome of mortality, anastomotic leak, and prolonged hospital stay were analyzed as outcome variables.

**Result:**

A total of 54 patients underwent esophagectomy for esophageal cancer during the study period. The mean age was 54 years. The mean duration of the surgery was 208 min. Intraoperative mean low SBP was 80 mmHg while the lowest record was 55 mmHg. IOH occurred in 51% (n = 29) of patients. Anastomotic leak occurred in 7% (n = 4) (OR 1.2, 95% CI 0.26–6.3; *p* = 0.76). In-hospital mortality was 5% (n = 3) (OR 1.44, 95% CI 0.22–9.3; *p* = 0.7) and 33% (n = 18) had prolonged hospital stay (OR 0.53, 95% CI 0.14–1.9; *p* = 0.34). The overall anastomotic leak rate was 13% (n = 7). Multivariate analysis (logistic regression model) showed SBP < 90 mmHg for more than 5 min was not significantly associated either with individual or composite outcomes of mortality, anastomotic leak, and prolonged hospital stay (AOR 1.06, 95% CI 0.98–1.14; p = 0.16)

**Conclusion:**

In patients undergoing esophagectomy for esophageal cancer, a systolic blood pressure < 90 mm Hg for greater than 5 min during surgery has no significant statistical association with composite adverse outcomes of mortality, anastomotic leak, and prolonged hospital stay.

## Background

Globally esophageal cancer is on the rise being the 8th most common cancer and 6th most common cause of death from cancer [1]. It is, particularly, increasing in Sub-Saharan Africa with higher incidence rates in Eastern and Southern African Sub-regions [2]. It is also the leading cause of elective cardiothoracic admissions to the surgical ward for procedures performed at our University hospital [3]. Esophageal cancer results in severe multifaceted insults to the physiology and cardiorespiratory reserve. Not only does it cause homeostatic derangements due to cachexia as in other malignancies, but the dysphagia and subsequent dehydration further confound their clinical status [4]. The presenting symptoms of esophageal cancer usually signify locally advanced disease, distant metastases, or both, irrespective of histologic type; even in cases with “early” diagnosis [5, 6]. Hence, patients tend to have an overall poor performance status, a state of depleted intravascular volume, and hypoalbuminemia at the time of diagnosis resulting in a higher risk of postoperative morbidity and mortality.

Esophagectomy, the mainstay of management in esophageal cancer, is a major and complex surgery involving the abdomen, chest, and/or neck. It is commonly associated with significant blood loss [7–10]. Manipulation of the mediastinum during surgery often leads to decreased preload, vagal stimulation, and arrhythmia [11, 12], thereby worsening the state of hypovolemia and hypoperfusion. It is estimated up to 75% of esophagostomies are associated with intraoperative hypotension [13]. There is, however, a paucity of data on the relationship of intraoperative low Systolic Blood Pressure (SBP) and postoperative outcomes during esophagectomy particularly from low resource, high volume centers.

We, therefore, prospectively studied the effect of intraoperative hypotension during esophagectomy and adverse short-term postoperative outcomes among patients operated for esophageal cancer.

## Methods

### Study design and data collection

This is a prospective observational cohort study on patients who underwent esophagectomy for esophageal cancer from August 1, 2017, to March 30, 2020. Diagnosis was based on clinical presentation; endoscopic evaluation, biopsy, and radiologic study (contrast x-rays and/or CT scan).

### Inclusion criteria

All male and female patients older than18 years of age who underwent standard esophagectomy were included in the study. Standard esophagectomy is defined as a subtotal resection of the esophagus which is reconstructed using one of the following conduits stomach, colon, or jejunum with one of the following four surgical approaches: trans hiatal, transthoracic (Ivor-Lewis), McKeown's esophagectomy or left thoracotomy.

### Exclusion criteria

Patients with poor pre-operative performance status, patients with cervical esophageal cancer, and patients who had signs indicative of advanced disease state such as hoarseness of voice, malignant ascites …etc. were excluded.

### Sample size and sampling technique

The sample size was calculated using statistical software Epinfo with a power of 80 and a CI of 95%. Consecutive patients used in sampling technique.

### Data collection and data collection tool

Data collected prospectively in a structured and pretested data collection format. Socio-demographics, clinical information on preoperative and intraoperative variables, as well as postoperative morbidity, mortality, and post-op stay documented. Modified Takita’s grading 1–6 [14] was used for the assessment of dysphagia. American Society of Anesthesiology (ASA) physical status classification system I–IV [15] for preoperative anesthesiology evaluation, BMI (body mass index) based on body weight and height (kg/m^2^), Eastern Cooperative Oncology Group (ECOG) performance classification (0–4) [16], serum albumin, serum creatinine, serum electrolytes, and liver enzyme tests were recorded. Preoperative ECG, echocardiography, and radiologic characteristics from barium swallow and CT studies were also recorded. AJCC 8th edition Esophageal Cancer staging was used for clinical staging [17].

### Definitions

As one of the frequently used thresholds identified in a systematic review done by Bijker et al [18], SBP < 90 mmHg, and duration of more than 5 min [18, 19] was used to define Intra Operative Hypotension (IOH).

Anastomotic leak was defined by a clinically diagnosed leak, and prolonged hospital stay was defined as hospital stay more than 7th post-op day.

Intraoperative blood loss, intraoperative events including arrhythmias, need for blood transfusion, need for inotropic, and/or vasopressor support were documented.

Trans Hiatal esophagectomy was preferred for mid and distal thoracic esophageal cancers and performed in 51% (n = 26) of cases. McKeown’s esophagectomy was preferred for mid and upper thoracic esophageal cancers which are at T4 stage and performed in 16% (n = 8) of the cases. Ivor–Lewis procedure was performed for 4% (n = 2) patients while 30% (n = 16) patients had Left thoracotomy approach as it was preferred for gastroesophageal junction and proximal gastric cancers. All esophagogastric anastomosis (stomach was used as a conduit in all cases) was done via the anatomical esophageal bed and with hand-sewn techniques.

### End point

The primary endpoint was the composite outcome of anastomotic leak, mortality of any cause, and prolonged postop hospital stay. Patients were followed for 30 days post-operatively.

### Data quality assurance

Data completeness checked by reviewing data collection format and patient medical records regularly.

### Ethical consideration

An approval from the Institutional ethics review board (Addis Ababa University College of Health Sciences: Protocol Number 084/17/Surg.) was acquired and written consent was obtained from the patients.

### Statistical analysis

IBM SSPS 23 software package used for statistical data analysis. Descriptive statistics used for describing the data, results are presented in percentage, and simple frequency, mean (SD), and median were used for other data. Factors with a possible influence on perioperative morbidity and mortality were calculated using multivariate regression analysis. A *p* value of < 0.05 was considered statistically significant.

## Results

### Socio-demographic characteristics

A total of 54 patients were included in the study. Mean age was 54 years (SD ± 12.08) and 61% (n = 33) were females. The mean body weight of the study participants was 49.04 (SD ± 9.74) Kg and the mean BMI was 18.6 (SD ± 2.85). Thirty-three (62%) of the patients were from a rural area and 30% (n = 16) of the study participants were from esophageal cancer endemic localities of the country (Table 1).

**Table 1:** Socio-demographic characteristics of the study participants

Variable	Number (%)
Sex	
Male	21 (39)
Female	33 (61)
Residence	
Urban	20 ( 38)
Rural	34 (62)

### Clinical presentation

Fifty patients (93%) presented with a chief complaint of dysphagia and the mean duration of dysphagia was 7 months (SD ± 0.5.2). Grade III and IV dysphagia were more common presentations than other grades of dysphagia 32% (n = 17) and 35% (n = 19) respectively. No patient had pre-operative neoadjuvant treatment. No supraclavicular lymph node was appreciated clinically in 93% (n = 50) cases at presentation (Table 2).

**Table 2:** Clinical parameters

Variable	Number (%)
Presence of dysphagia	
Yes	50 (93)
No	4 (7)
Degree of dysphagia	
Complains but can still swallow (I)	1 (2)
Requires liquid to swallow (II)	6 (11)
Can swallow semisolids but not solids (III)	17 (32)
Can swallow liquids but not semisolids (IV)	19 (35)
Can swallow saliva but not semisolids (V)	9 (17)
Can’t even swallow saliva (VI)	2 (4)
Presence of supraclavicular lymph node (LN)	
Yes	2 (4)
No	50 (92)
Missed data	2 (4)

### Preoperative risk assessment

Fifty-two (96%) of the patients were in a good performance state with ECOG class 2 or less. There was no patient in ECOG class 4. Fifty percent (n = 27) were in ASA class 1 classification and 89% (n = 48) had no known comorbidity. Cardiovascular disease (mostly hypertension) was the commonest comorbidity found in 4 (7%) of patients. Only 7% (n = 4) had a history of smoking.

### Investigation results

Mean preoperative hematocrit was 38.41% (SD ± 8.14) and mean WBC was 6674.2 (SD: ± 2058). Mean serum albumin was 3.92 (SD: ± 0.85) g/dl and median serum K^+^ was 4.00 (IQR 3.60–4.50) meq /L. Mean serum creatinine was 1.04 mg/dl and 89% (n = 48) patients had no derangement of liver enzyme tests. Minor abnormal ECG was noted in 23% (n = 12) patients and 8% (n = 4) cases had evidence of old ischemic changes.

CT imaging was done for 86% (n = 44) of the cases 24% (n = 10) and 4.7% (n = 2) had a loss of fat plane between the aorta and esophagus and between the tracheobronchial tree and esophagus respectively. Mean length of malignant strictures on barium swallow study and CT imaging was 4.94 (SD ± 2.11) cm. Fifty-two (96%) had upper GI Endoscopy evaluation. The mean location of the tumor from incisors was at 32 cm (SD: ± 4.67). Biopsy results have revealed Squamous cell carcinoma in 72% (n = 39) and Adenocarcinoma in 19% (10). There were 9% (5) patients with no Biopsy result.

### Intraoperative findings and events

Thirty-nine (72%) patients were in clinically stage III disease and 9% (n = 5) patients were in stage IV. More than 90% of the patients were found to have T3 or advanced tumor stage during surgery. Three (6%) patients had an invasion of unresectable structures such as the aorta. All patients had lymph node involvement with N1 and N2 stage involvements being the commonest. Five patients had signs of gross metastasis. Omental wrap was used in 51% (n = 24) of the cases.

Mean duration of surgical procedures was 208.6 min (SD ± 65.89) and the mean duration of anesthesia was 238 min (SD ± 75.65). Mean estimated blood loss was 741 ml and the median estimated blood loss of the procedure was 500.00 (IQR: 300.00–695.00) ml. One (2%) patient who had significant bleeding of approximately 1000 ml. Mean total duration of SBP < 90 mmHg was 18.3 (SD ± 28.5) minutes. Mean SBP during IOH was 80 mmHg (SD ± 12.4) while the lowest record was SBP of 55 mmHg. The median lowest SBP was 80.00 (IQR: 73.50–90.00) mmHg. IOH occurred in 51% (n = 29) of the time. Most IOH episodes were corrected by boluses of crystalloids infusion. IOH occurring due to mediastinal manipulations resolved with an intermittent withdrawal of the surgeon's hand. Ten (19%) patients needed an intraoperative blood transfusion and ten patients (19%) required intraoperative inotropic or vasopressor support. Epinephrine and Phenylephrine were inotropic and vasopressor support drugs of choices. No patient had an epidural catheter in place (Table 3.)

**Table 3:** Intraoperative findings and events

Variable	Number (%)
Clinical (intra operative) tumor staging	
T stage	
T1 (invasion of submucosa)	0 (0)
T2 (invasion of Muscularis propria)	5 (9)
T3 (invasion of adventitia)	25 (46)
T4A (invasion of resectable adjacent structures)	21 (39)
T4B (invasion of unresectable adjacent structures)	3 (56)
N stage	
N0 (no LN invasion)	0 (0)
N1 (1–2 regional LN involvement)	19 (35
N2 (3–6 regional LN involvement)	23 (43)
N3 (≥ 7 regional LN involvement)	12 (22)
M stage	
M0 (no metastasis)	49 (91)
M1 (distant metastasis)	5 (9.)
Ascites	2 (4)
Liver metastasis	2 (4)
Lung metastasis	1 (2)
Clinical stage	
I	0 (0)
II	10 (19)
III	39 (72)
IV	5 (9)
Omental wrap use	
Yes	24 (44)
No	23 (43)
Missing data	7 (13)
Operative complications^a^	
Tumor perforations	3 (5.5)
R2 resection	3 (5.5)
Chylothorax	1 (2)
Recurrent laryngeal nerve injury	1 (2)
Others^b^	4 (7)
No complication	42 (78)
Need for intraoperative blood transfusion	
Yes	10 (19)
No	43 (81)
Missing data	1 (2)
Need for intraoperative inotropic support	
Yes	15 (28)
No	32 (59)
Missing data	7 (4)

^a^Includes complications observed both intra op and post-op

^b^Includes persistent air leak from right, pleural breach, pyothorax, splenic injury (splenectomy) gall bladder injury (cholecystectomy)

### Postoperative care and outcomes

Most Patients 45 (83%)were extubated on the OR table and observed in a Post Anesthetic Care Unit for the first 6hrs immediate post-op before deemed stable enough to be transferred to the general ward. Nine Patients (17) patients were transferred to ICU and subsequently extubated the next immediate hours to the first post-op day. Ten patients (19%) who needed inotropic and or vasopressor support were transferred to the ICU until they were weaned off inotropic and or vasopressor support and deemed stable enough to be transferred to their respective wards as well.

The 30 days operative mortality was 9% (n = 5). Among the in-hospital deaths, 4% (n = 2) were attributed to anastomotic leak. One (2%) patient died after discharge within the study period of 30 post-op days from an unknown cause. There were 13% (n = 7) cases of anastomotic leak. Six (12%) patients underwent reoperations such as feeding jejunostomy tube insertion for complications (anastomotic leak). Mean post-op hospital stay was 12 days. Thirty (55%) of patients had a prolonged hospital stay. Fifteen (29%) and 5 (10%) patients needed a postoperative blood transfusion and postoperative inotropic support respectively. On 30th-day post-op 57% (n = 31) of the patients were ambulatory in more than 50% of waking hours and capable of all self-care (ECOG 3) (Table 4).

**Table 4:** Postoperative care and outcomes

Variable	Number (%)
Need for postoperative blood transfusion	
Yes	15 (28)
No	37 (68)
Missing data	2 (4)
Need for postop inotropic/vasopressor support	
Yes	5 (9)
No	47 (87)
Missing data	2 (4)
Anastomotic leak	
Yes	7 (13)
No	47 (87)
Reoperation for complication	
Yes	6 (11)
No	48 (89)
30 days mortality	6 (11)
In hospital	5 (9)
Post-discharge, within 30 days post-op	1 (2)
Probable cause of death attributed to anastomotic leak	2 (4)
Probable cause of death not attributed to anastomotic leak	
MI	1 (2)
Stroke	1 (2)
Chylothorax (sepsis, hypotension)	1 (2)
ECOG performance status 30th postoperative day	
0: Fully active	2 (4)
1: Restricted in physically strenuous activity but ambulatory	10 (19)
2: Ambulatory and capable of all self-care but unable to carryout any work activities; up and about > 50% of waking hours	19 (35)
3: Capable of only limited self-care; confined to a bed or chair > 50% of waking hours	18 (33)
4:Completely disabled; cannot carry on any self-care; totally confined to bed or chair	0 (0)
Missing data^a^	4 (7)

^a^Excluding one death post-discharge

### Outcome of patients and associated factors

Four (7%) patients with anastomotic leak (OR 1.2, 95% CI 0.26–6.3; *p* = 0.76), 3 (5%) patients who died (OR 1.44, 95% CI 0.22–9.3; *p* = 0.7) and 18 (33%) with prolonged hospital stay (OR 0.53, 95% CI 0.14–1.9 *p* = 0.34) had experienced IOH (Table 5).

**Table 5:** Intraoperative hypotension and outcome variables

Endpoint	No intra op hypotension N (%)	Intra op hypotension N (%)	OR 95% CI	p value
Hospital stay				
≤ 7 days	5 (9)	7 (13)	0.53 (0.14–1.9)	*p* = 0.34
> 7 days	24 (44)	18 (33.3)		
Anastomotic leak				
Yes	3 (5)	4 (7)	1.28 (0.26- 6.3)	*p* = *0.76*
No	23 (43)	24 (44)		
Death				
Yes	2 (4)	3 (5)	1.44 (0.22–9.3)	*p* = 0.70
No	24 (44)	25 (46)		

Multivariable binary logistic regression analysis showed SBP < 90mmHG for > 5 min was not significantly associated with composite outcomes of anastomotic leak, mortality, and prolonged hospital stay (AOR 1.06, 95% CI 0.98–1.14; *p* = 0.16)*.*

Patients who had N3 (≥ 7 LN) clinical intraoperative tumor stage were 96% less likely to have good composite outcome compared to those patients who had N1 (< 3 LN) clinical intraoperative tumor stage (AOR 0.04, 95% CI 0.01–0.97; *p* = 0.048) (Table 6).

**Table 6:** Composite outcome and perioperative factors

Variables	COR (95% CI)	p value	AOR^a^ (95% CI)	p value
N stage				
N1 (< 3 LN)	1		1	
N2 (3–7 LN)	0.53 (0.13, 2.23)	0.388	0.27 (0.05, 1.43)	0.125
N3 (> 7 LN)	0.13 (0.12, 1.01)	0.051	0.04 (0.01, 0.97)	0.048*
SBP < 90 mmHg > 5 min	1.06 (0.99, 1.12)	0.056	1.06 (0.98, 1.14)	0.160
SBP < 90 mmHg	2.36 (0.7, 7.93)	0.166	1.07 (0.16,6.99)	0.945
Lowest SBP	0.96 (0.91, 1.01)	0.135	0.98 (0.93, 1.03	0.423
Pre op ECOG performance				
Level 0 and 1	0.41 (0.11, 1.51)	0.181	0.46 (0.08, 2.73)	0.394
Level 2 and 3	1		1	

^*^Statistically significant

^a^AOR: adjusted for mortality, anastomotic leak, and prolonged hospital stay

## Discussion

The rates of morbidity and mortality following esophagectomy for esophageal cancer are improving [20]. In a 1980 review article, operative mortality for esophageal resection was 29% [20]. In mid-2000s operative mortality decreased to 10–11% [9, 21, 22]. While multiple works of literature suggested tumor stage, histologic subtype, performance status, age, type of surgical approach, intraoperative blood loss, and blood transfusion as risk factors, few have clearly addressed the effect of intraoperative hypotension on postoperative morbidity and mortality of patients undergoing esophagectomy for esophageal cancer [9, 10, 21–27]. Furthermore, the lack of agreement on the definitions of intraoperative hypotension (IOH) during surgical procedures, including esophageal resections has confounded the association between blood pressure deviations during surgery and mortality [15, 18]. The paucity of such studies makes a comparison with our study challenging.

In our study, we found that neither intraoperative hypotension, SBP < 90 mm Hg for more than 5 min (OR 1.06, 95% CI 0.98–1.14; *p* = 0.160) nor the lowest SBP (OR 1.07, 95% CI 0.16- 6.99; *p* = 0.945) were associated with adverse composite outcomes of mortality, anastomotic leak or prolonged hospital stay. The overall mortality was 9% while 3 (5%) of deaths are associated with IOH. This was similar to post esophagectomy mortality rates of 3–16% reported by multiple studies [9, 10, 21–23, 25]. In this study mortality adjusted for trans hiatal esophagectomy (THE) only, was 10% which was less than the 18.7% reported in a 2012 study for THE in the same institution. [7]

IOH was not found to be associated with an increased perioperative mortality (5.5% vs 3.3%; OR 1.44 (0.22, 9.3) *p* = 0.7). This finding aligns with the retrospective cohort study on combined intraoperative blood pressure data by Monk et al [19] which identified Systolic BP < 70 mmHg, not higher, for ≥ 5 min to be associated with increased 30-day operative mortality in non-cardiac surgery. A study by Fujisawa. et al [26] found that patients with intraoperative hypotension showed significantly lower 1-year cancer-specific survival than patients without hypotensive episodes (*p* = 0.0002). They, however, defined intraoperative hypotension as SBP < 70 mmHg and did not describe the short-term outcome.

Anastomotic leak occurred in overall 7 patients (13%) and 4 (7%) in patients with IOH. There were no leaks from the left thoracotomy approach with left intrathoracic anastomosis while 1 patient leaked from right intrathoracic anastomosis while the remaining 6 patients had a cervical leak. As it is well known that cervical anastomosis leak rates are higher due to the tension applied to the stomach to reach the neck. This further explains why left thoracotomy approaches are very unlikely to have leaks because the tension on the stomach is very less followed by right intrathoracic anastomosis.

The anastomotic leak we found in this study had no significant statistical association with intraoperative hypotension (6% vs 7%; *p* = 0.76). This is in contrast to the finding by Fumagall et al. [27] where leaks were significantly more common in patients with intra-operative hypotensive episodes (*p* = 0.02). Their study involved a larger patient number (84), defined hypotensive episodes as SBP decreasing more than 30% of the basal value for more than 5 min, and had procedures performed in a prone position. Unlike their study, none of our study patients was operated on in a prone position.

Our anastomotic leaks accounted for 2 (4%), of the deaths and had a 2/7 (28%) mortality which is comparatively higher than a 12% mortality from anastomotic leak found in a systematic review done by Verstegen et al. [28] and other recent data [30] but comparable to the 37% mortality reported by Turkyilmaz et al. [29].

In 3 cases there was part of a tumor leftover as it was deemed dangerous to proceed with complete tumor removal due to the T4b stage of the tumor. These were not avoided from preoperative work due to a combination of pre-operative radiologic imaging’s' failure to properly identify the stage of the tumor, the fact that there are very few oncologic units which are overwhelmed by oncologic patients and the lack of esophageal stenting for palliation. Hence as an institution, we opt to operate/surgically explore patients unless they have clear evidence of advanced disease identified preoperatively.

Even though Gockel and colleges [9] in their study involving 424 patients suggested that tumor characteristics, e.g. TNM classification, were of no influence on the postoperative course our study, however, found that N3 stage, hence stage III disease, is significantly associated with adverse short term postoperative outcomes (AOR 0.04 (0.01–0.97), *p* = 0.048). This result is in agreement with other risk analysis studies, which suggest that those with stage III or IV disease have a higher postoperative mortality [30, 31].

### Limitations and recommendations

In this study, we have identified certain limitations. It has a small sample size and has some missing data. The study also has not addressed the effect of sustained and non-sustained IOH. Additionally, IOH was not defined and analyzed in terms of mean arterial and diastolic blood pressure on short-term post-op outcomes. Moreover, the study did not analyze the association and outcome of IOH with stage subtypes, different esophagectomy approaches, and histologic subtypes. Furthermore, the study has not addressed other secondary endpoints such as wound infection, pulmonary complications…etc.

## Conclusion

In this study, we found that a systolic blood pressure < 90 mmHg for greater than 5 min during surgery has no significant statistical association with composite adverse outcomes of mortality, anastomotic leak, and prolonged hospital stay.

## Data Availability

The datasets used and/or analyzed during the current study are available from the corresponding author on reasonable request.

## Abbreviations

AJCC: American Joint Committee on Cancer
ASA: American Society of Anesthesiology
AOR: Adjusted odds ratio
BMI: Body mass index
CI: Confidence interval
COR: Crude odds ratio
ECG: Electrocardiogram
ECOG: Eastern Cooperative Oncology Group
IOH: Intraoperative hypotension
LN: Lymph node
OR: Odds ratio
RBBB: Right bundle branch block
SBP: Systolic blood pressure
TNM: Tumor lymph node metastasis
THE: Trans hiatal esophagectomy

## References

[1] https://globocan.iarc.fr/Pages/fact_sheets_cancer.aspx.

[2] Rabson K (2010). Systematic review: epidemiology of oesophageal cancer in Sub Saharan Africa. Malawi Med J..

[3] Ephraim T, Seyoum K, Adem A (2013). Patterns of cardiothoracic admissions at a tertiary hospital in Ethiopia. East Cent Afr J Surg.

[4] Poorna A, Pernilla L (2016). Cachexia in patients with oesophageal cancer. Nat Rev Clin Oncol.

[5] Orringer MB, Marshall B, Iannettoni MD (2001). Transhiatal esophagectomy for the treatment of benign and malignant esophageal disease. World J Surg.

[6] Jemal A, Murray T, Samuels A, Ghafoor A, Ward E, Thun M (2003). Cancer statistics, 2003. CA Cancer J Clin.

[7] Alemu BN, Ali A, Gulilat D, Kassa S, Bekele A (2012). Outcome of trans hiatal esophagectomy done for advanced oesophageal cancer. East Cent Afr J Surg..

[8] Orringer MB, Marshall B, Chang A, Lee J, Pickens A, Lau CL (2007). Two thousand transhiatal esophagectomies changing trends, lessons learned. Ann Surg..

[9] Gockel I, Exner C, Junginger T (2005). Morbidity and mortality after esophagectomy for esophageal carcinoma: a risk analysis. World J Surg Oncol..

[10] Whooley B, Law S, Murthy S, Alexandrou WJ (2001). Analysis of reduced death and complication rates after esophageal resection. Ann Surg.

[11] Sepesi B, Swisher SG, Walsh GL, Correa A, Mehran RJ, Rice D (2012). Omental reinforcement of the thoracic esophagogastric anastomosis: an analysis of leak and reintervention rates in patients undergoing planned and salvage esophagectomy. J Thorac Cardiovasc Surg.

[12] Daly JM, Karnell LH, Menck HR (1996). National cancer database report on esophageal carcinoma. Cancer.

[13] Nikbakhsh N, Amri P, Shakeri A, Shakeri A (2012). Changes in blood pressure and heart rhythm during transhiatal esophagectomy. Caspian J Intern Med..

[14] Takita H, Vincent R, Caicedo V, Gutierrez A (1977). Squamous cell carcinoma of the esophagus: a study of 153 cases. J Surg Oncol..

[15] https://www.asahq.org/standards-and-guidelines/asa-physical-status-classification-system.

[16] https://ecog-acrin.org/resources/ecog-performance-status.

[17] Rice T, Patil DT, Blackstone E (2017). 8th edition AJCC/UICC staging of cancers of the esophagus and esophagogastric junction: application to clinical practice. Ann Cardiothorac Surg..

[18] Bijker J, van Klei W, Kappen T, Wolfswinkel L, Moons K, Kalkman C (2007). Incidence of intraoperative hypotension as a function of the chosen definition: literature definitions applied to a retrospective cohort using automated data collection. Anesthesiology.

[19] Monk T, Bronsert M, Henderson W, Mangione M, Sum­Ping ST, et al. Association between intraoperative hypotension and hypertension and 30-day postoperative mortality in noncardiac surgery. Anesthesiology. 2015;123(8); 307–19. 10.1097/ALN.0000000000000756 26083768

[20] Earlam R, Cunha-Melo J (1980). Esophageal squamous cell carcinoma. A critical review of surgery. Br J Surg..

[21] D’Amico TA (2007). Outcomes after surgery for esophageal cancer gastrointestinal cancer. Gastrointest Cancer Res..

[22] Atkins BZ, Shah AS, Hutcheson KA, Mangum J, Pappas T, Harpole D (2004). Reducing hospital morbidity and mortality following esophagectomy. AnnThorac Surg.

[23] Bailey S, Bull D, Harpole D, Rentz J, Neumayer L, Pappas T (2003). Outcomes after esophagectomy. Ann Thorac Surg.

[24] Law SYK, Fok M, Wong J (1994). Risk analysis in resection of squamous cell carcinoma of the esophagus. World J Surg.

[25] Mariette C, Taillier G, Seuningen IV, Triboulet JP (2004). Factors affecting postoperative course and survival after en bloc resection for esophageal carcinoma. Ann Thorac Surg.

[26] Fujisawa A, Yamauchi-Satomoto M, Uchida T, Miyawaki Y, Kawano T, Makita K (2012). Potential influence of intraoperative hypotensive episodes on postoperative recurrence and survival in patients with complete resection of esophageal cancer. Eur J Anaesthesiol..

[27] Fumagalli U, Melis A, Balazova J, Lascari V, Morenghi E, Riccardo RR (2016). Intra-operative hypotensive episodes may be associated with post-operative esophageal anastomotic leak. Updates Surg.

[28] Verstegen M, Bouwense S, van Workum F, ten Broek R, Siersema P, Rovers M, Rosman C, et al Management of intrathoracic and cervical anastomotic leakage after esophagectomy for esophageal cancer: a systematic review. World J Emerg Surg. 2019;14:17. 10.1186/s13017-019-0235-4. 10.1186/s13017-019-0235-4 PMC6449949 30988695

[29] Turkyilmaz A, Eroglu A, Aydin Y, Tekinbas C, Muharrem Erol M, Karaoglanoglu N (2009). The management of esophagogastric anastomotic leak after esophagectomy for esophageal carcinoma. Dis Esophagus..

[30] Lund O, Kimose HH, Aagaard MT, Hasenkam JM, Erlandsen M (1990). Risk stratification and long-term results after surgical treatment of carcinomas of the thoracic esophagus and cardia. J Thorac Cardiovasc Surg.

[31] Shao-bin C (2013). Prognostic factors and outcome for patients with esophageal squamous cell carcinoma underwent surgical resection alone. J Thorac Oncol..

